# Case-Based Serious Gaming for Complication Management in Colorectal and Pancreatic Surgery: Prospective Observational Study

**DOI:** 10.2196/44708

**Published:** 2023-11-09

**Authors:** Sophie-Caroline Schwarzkopf, Marius Distler, Thilo Welsch, Grit Krause-Jüttler, Jürgen Weitz, Fiona R Kolbinger

**Affiliations:** 1 Department of Visceral, Thoracic and Vascular Surgery, University Hospital and Faculty of Medicine Carl Gustav Carus, TUD Dresden University of Technology Dresden Germany; 2 National Center for Tumor Diseases (NCT/UCC), Dresden, Germany; German Cancer Research Center (DKFZ), Heidelberg, Germany; Faculty of Medicine and University Hospital Carl Gustav Carus, TUD Dresden University of Technology, Dresden, Germany; Helmholtz-Zen Dresden Germany; 3 Department of General, Visceral, and Thoracic Surgery University Medical Center Hamburg-Eppendorf Hamburg Germany; 4 Centre for Tactile Internet with Human-in-the-Loop (CeTI), TUD Dresden University of Technology Dresden Germany

**Keywords:** Serious game, surgical education, clinical reasoning, medical education, postoperative complications, educational games, computer-based learning, colorectal surgery, decision-making, pancreatic surgery

## Abstract

**Background:**

The potential risk and subsequent impact of serious complications after pancreatic and colorectal surgery can be significantly reduced through early recognition, correct assessment, and timely initiation of appropriate therapy. Serious gaming (SG) is an innovative teaching method that combines play with knowledge acquisition, increased concentration, and quick decision-making and could therefore be used for clinically oriented education.

**Objective:**

This study aims to develop a case-based SG platform for complication management in pancreatic and colorectal surgery, validate the application by comparing game courses of various professional groups in the health care sector, and test the acceptance of the developed platform in the context of clinical education by measuring levels of usability and applicability within the framework of a validity and usefulness analysis.

**Methods:**

In this observational trial, a novel SG for management of postoperative complications was developed and prospectively validated in a cohort of 131 human caregivers with varying experience in abdominal surgery. A total of 6 realistic patient cases were implemented, representing common complications after pancreatic and colorectal surgery. Cases were developed and illustrated using anonymized images, data, and histories of postoperative patients. In the prospective section of this study, following a brief case presentation, participants were asked to triage the virtual patient, make an initial suspected diagnosis, and design a 3-step management plan, throughout which the results of selected diagnostic and therapeutic actions were presented. Participants’ proposed case management was compared to ideal case management according to clinical guidelines. Usability, applicability, validity, and acceptance of the application were assessed using the Trier Teaching Evaluation Inventory as part of a noncomparative analysis. In addition, a comparative analysis of conventional teaching and learning formats was carried out.

**Results:**

A total of 131 cases were answered. Physicians selected more appropriate therapeutic measures than nonphysicians. In the Trier Teaching Evaluation Inventory, design, structure, relevance, timeliness, and interest promotion were predominantly rated positively. Most participants perceived the application to be superior to conventional lecture-based formats (training courses, lectures, and seminars) in terms of problem-solving skills (102/131, 77.9%), self-reflection (102/131, 77.9%), and usability and applicability (104/131, 79.4%).

**Conclusions:**

Case-based SG has educational potential for complication management in surgery and could thereby contribute to improvements in postoperative patient care.

## Introduction

Advanced inflammatory [1] or malignant [2,3] diseases of the pancreas or colorectum usually require surgical intervention. Unpreventable postoperative complications occur in up to every second abdominal surgery due to the severity and complexity of the procedure and patients’ general frailty [4,5]. Correct and rapid classification of symptoms is essential for the recognition and treatment of postoperative complications [6] such as postpancreatectomy hemorrhage [7], a potentially life-threatening complication after pancreatic surgery [8]. In such situations, misjudgments are directly associated with increased morbidity and mortality [2]. As the risk, severity, and subsequent impact of postoperative complications could be significantly reduced through early recognition, correct assessment, and timely initiation of appropriate therapy, the training of situation awareness and decision-making processes has the potential to translate into manifest clinical benefit [9].

In clinical practice, physicians face challenges related to rapidly changing health care systems and the need to constantly keep up with scientific advances [10]. Outdated doctrines can result in suboptimal care and medical errors [11]. Therefore, the establishment of new didactic formats can contribute to the maintenance of high standards, prove the effectiveness of innovative methods, and gain user acceptance [12,13]. Clinically oriented education aims to strengthen the theoretical, practical, and communication skills of medical staff and to prepare and motivate them for lifelong continuing training [14]. Various teaching methods with distinct characteristics can be combined to best achieve these goals [13,15]. Serious gaming (SG) is an innovative teaching method that combines play with knowledge acquisition, increased concentration, and quick decision-making [16,17]. The strengths of SG lie in the acquisition and subsequent transfer of procedural skills as well as declarative knowledge [18]. In addition, SG is suitable for teaching the peculiarities of individual cases, such as gender- or ethnicity-specific disease patterns, thus increasing awareness and raising consciousness. Recent evidence suggests that by implementing these aspects, both student education per se and the quality of patient care can be improved [19]. Traditionally, medical teaching takes place in person and offers relatively little interaction between students and teachers. During the COVID-19 pandemic, the need to digitize and restructure established formats has grown substantially, and both educators and students have realized that web-based teaching is not only feasible but also offers many advantages and opportunities, including independence in place and time and the promotion of cooperative learning [20]. Digital education options offer the potential to integrate recent evidence, contributing to lifelong learning and lasting knowledge. Considering the necessity not only to generate and make knowledge available but also to manage it, the potential of digital education has not yet been exhausted [21].

This project aims to develop and validate a scalable, case-based SG platform for clinical education of postoperative management targeting health care professionals throughout different stages of training. Ultimately, the aim is to improve the quality of care for hospitalized patients by increasing health care professionals’ capability to transfer knowledge to specific disease contexts and thus provide sensitized care.

## Methods

### Structure and Development of the SG

The SG was designed as an anonymized, prospective, open, observational study based on a voluntary web-based survey constructed with REDCap (Research Electronic Data Capture; Vanderbilt University) [22]. Participants included hospital staff with patient contact and medical students in German-speaking countries, and the study was carried out in German. A total of 6 independent SG cases were implemented. The cases represented complications after pancreatic surgery (postpancreatectomy hemorrhage or postoperative pancreatic fistula [POPF]), colorectal surgery (anastomotic leakage or mechanical ileus), and general postoperative complications (eg, stroke, wound infection, and COVID-19 infection). These 6 cases of postoperative complications were selected based on their rate of occurrence [23] and severity, as well as their potential to identify and respond to early clinical symptoms. Initial case selection took place with a drop-down menu that included brief case descriptions (ie, “4th postoperative day after ileostomy reversal, muscular defense” for the case on anastomotic leakage).

The SG platform allowed participants to plan and carry out diagnostic and therapeutic measures for fictitious postoperative patients who presented with new symptoms. After selecting 1 or more measures, participants were presented with the results of the respective diagnostic or therapeutic actions and could adjust their further actions accordingly. A total of 108 diagnostic and therapeutic measures, grouped into 16 measure categories, were available per selection level and case. Multimedia Appendix 1 gives an overview of measures and measure categories.

For case illustration, completely anonymized photographic images of visible disease symptoms or signs, laboratory parameters, imaging data, or other diagnostic tests of patients from the Department of Visceral, Thoracic, and Vascular Surgery at the University Hospital Carl Gustav Carus Dresden who underwent pancreatic or colorectal surgery between 2015 and 2021 were used.

**Figure 1:**
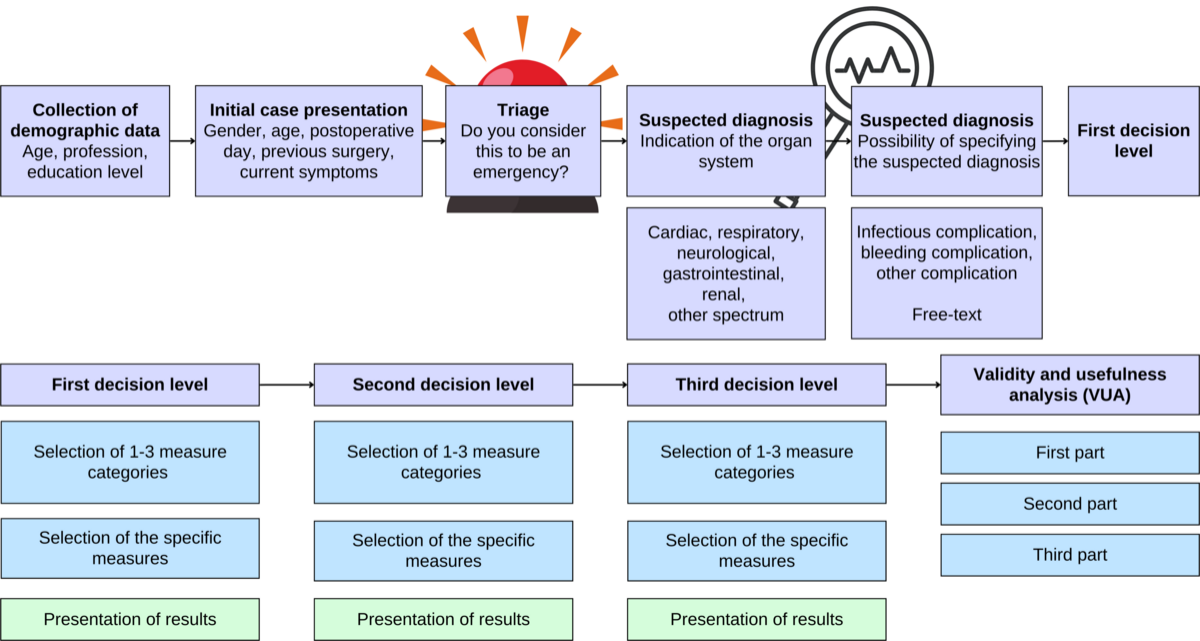
Serious gaming case structure. Introductory part: from the collection of demographic data of the participant to the first decision level. Main part includes 3-step case management and validity and usefulness analysis.

For general statistical evaluations, demographic data were collected at the beginning of each game case. The following initial case presentation provided patient information. Subsequently, participants were asked to make a suspected diagnosis and select initial diagnostic and therapeutic measures from a list. The measure results were presented directly after selection. This was followed by 2 more decision levels, with the first level representing the acute, the second the definitive, and the third the long-term management of the suspected complication (Figure 1).

Between levels, participants were asked to change or narrow down their current suspected diagnosis, mandatorily indicating a suspected affected organ system and offering further specification of the complication mechanism and the complication itself. Furthermore, it was recorded whether the participants considered the case at hand to be an emergency (binary question). Each case was concluded with a validity and usefulness analysis (VUA).

### VUA Framework

The first part of the VUA was based on the Trier Teaching Evaluation Inventory (Figure 1). The second part allowed a comparison of the application to existing lecture-based teaching and learning. The third part enabled an evaluation of the expected knowledge gain in comparison with common theoretical or practical learning formats. Finally, the case was to be evaluated with a grade (1=best to 6=worst grade).

All answers were given by means of a fully verbalized, 5-point, balanced scale (eg, scale end points of the first part: “agree,” “rather agree,” “undecided,” “rather disagree,” and “disagree”; second and third part: “SG is superior,” “SG is rather superior,” “undecided,” “SG is rather inferior,” and “SG is inferior”).

### Participant Recruitment

Pilot-testing and evaluation of the SG took place from December 2021 to February 2022. We aimed to recruit a diverse cohort of health care professionals as potential users of our application. To this end, the SG was advertised through various channels, including direct email advertisements to medical and nursing staff of the University Hospital Carl Gustav Carus Dresden and students of the Medical Faculty Carl Gustav Carus Dresden, web-based portals of the Technical University Dresden, and direct information events. Participants not affiliated with the University Hospital Dresden were reached by email through appropriate mailing lists (eg, mail distribution lists of German clinics). Participants were recruited from the entire German-speaking area, and the study was carried out in German.

The prerequisite for participation was the ability to give informed consent and the informed consent itself. Only fully answered cases were included in the final analysis.

### Ethical Considerations

This study was carried out in accordance with the Declaration of Helsinki and its later amendments. The Ethics Committee of the Technical University Dresden reviewed and approved this study (BO-EK-378072021). Before participation, all participants consented to anonymized data acquisition, data analysis, and publication of the results.

### Game Process Evaluation and Statistical Analysis

The SG case itself and the subsequent VUA were evaluated separately. The general statistical evaluation comprised a descriptive evaluation of the participant cohort, an evaluation of processing times, and a comparative evaluation of differences in case duration and duration of processing time of individual management levels between occupational and educational groups using ANOVA. Initial and final suspected diagnoses were evaluated using descriptive statistics, with free-text statements classified as “correct,” “incorrect,” and “not specified.” The results were displayed using frequency distributions by case and occupational group.

In order to analyze the appropriateness of management decisions, an ideal solution path was elaborated for each case, corresponding to a specific sequence of diagnostic and therapeutic measures. Besides this ideal solution path, 1 measure was defined as crucial for each SG case, that is, essential for the recovery of the fictitious patient, by 2 surgical experts with over 15 years of clinical experience in general and abdominal surgery (TW and MD). This therapeutic action was used to assess appropriate, exaggerated, and understated therapy escalation. The appropriateness of the selected therapeutic pathway was rated based on all clinical decisions made throughout the course of the SG case. These evaluations were based on valid German or European guidelines [7,24-26], expert opinions, and publications, as well as existing evidence on complication management in abdominal surgery, to ensure medical correctness and contextual coherence as well as a straightforward and realistic initial presentation. The ideal paths with the appropriate therapeutic measures for all cases as well as initial case presentations are presented in Multimedia Appendix 2, and measure categories and individual measures are presented in Multimedia Appendix 1. Final diagnoses had to be given in free-text format.

VUA results were summarized using descriptive statistics. Raw data were exported directly from REDCap [22] and are provided in Multimedia Appendix 3. The subsequent statistical analysis and illustration were performed using R software (R Foundation for Statistical Computing) [27], GraphPad Prism (version 9; Dotmatics) [28], and Canva (Canva Inc) [29]. Significance in groupwise comparisons was assessed using ANOVA (for numerical variables) and the Fisher exact test (for categorical variables), with *P*<.05 considered statistically significant.

## Results

### Participant Cohort and Processing Time

A total of 131 SG cases were completed in the desktop-based SG application (Multimedia Appendix 4). Overall, the participant cohort reflected the entire spectrum of health care professionals with the largest proportions; 57% (74/131), 15% (20/131), and 14% (18/131) of cases were completed by medical students, nursing and other clinical staff with patient contact, and experts, respectively (Multimedia Appendix 5). The age group of 21-25 years was most frequently represented (45/131, 34%), and the average age of the participants was 30.8 (SD 10.4) years (Multimedia Appendix 4). On average, a single SG session took 11 minutes and 46 seconds. No significant differences regarding the duration were found between cases (*P*=.68; Multimedia Appendix 6), age (*P*=.46; Multimedia Appendix 4), or occupational groups (*P*=.33; Multimedia Appendix 5).

In addition, a cross-case analysis was performed comparing the time needed per measure stage, which was defined from the beginning of the selection of the measure top categories per stage until the presentation of the results of the respective stage. The analysis showed a significant difference in processing time. On average, participants needed 1 minute and 25 seconds for the first stage, 53 seconds for the second stage, and 30 seconds for the third stage (all *P*<.001; Table 1).

**Table 1:** Average duration of serious gaming cases with respect to management level.

3-step case management	Average duration	*P* value
Acute management	1 min 25 s	<.001
Definitive management	53 s	<.001
Long-term management	30 s	<.001

### SG Cases Discriminate Between Professions and Surgical Expertise

**Figure 2:**
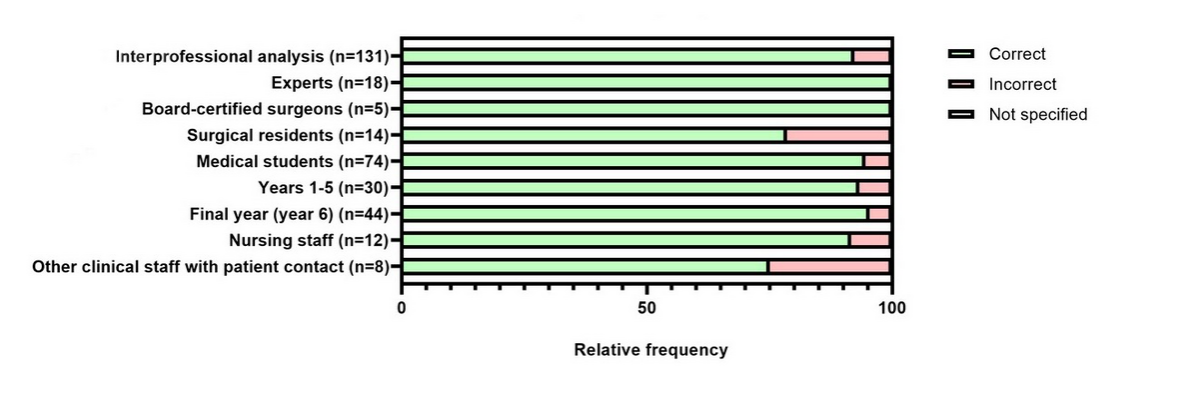
Interprofessional evaluation of the initially specified suspected diagnosis by suspected organ system across all serious gaming cases. Specification of the organ system was mandatory, while specification of complication mechanism and exact complication were voluntary.

**Figure 3:**
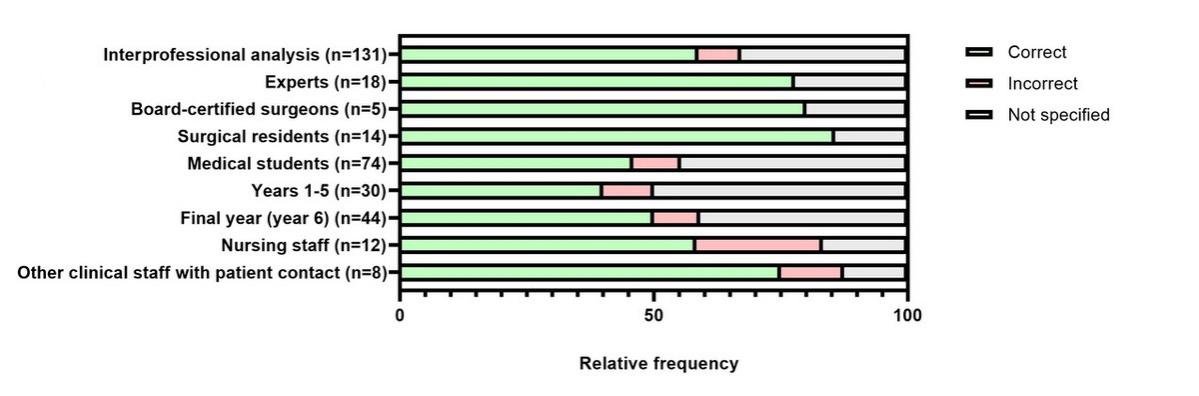
Interprofessional evaluation of the initially specified suspected diagnosis by suspected complication mechanism across all serious gaming cases. Specification of the organ system was mandatory, while specification of complication mechanism and exact complication were voluntary.

**Figure 4:**
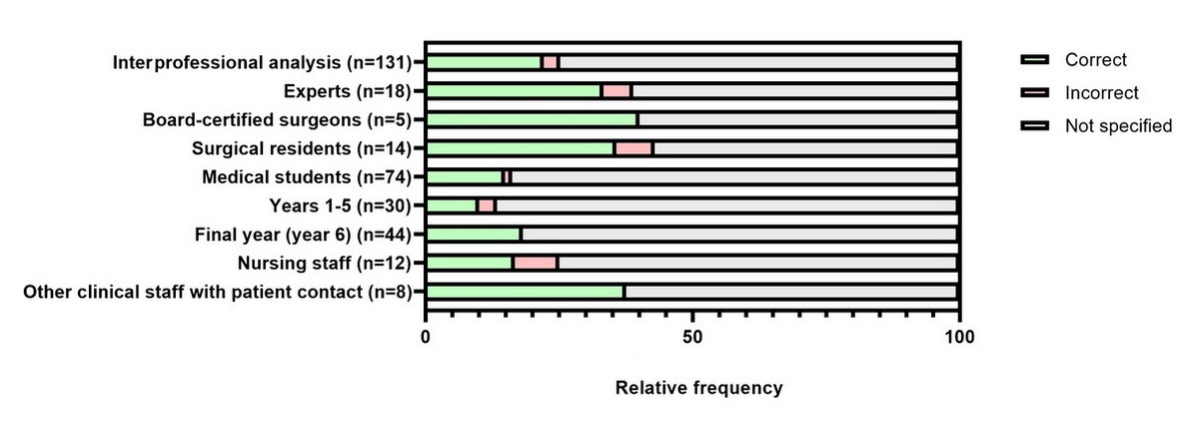
Interprofessional evaluation of the initially specified suspected diagnosis by suspected exact complication across all serious gaming cases. Specification of the organ system was mandatory, while specification of complication mechanism and exact complication were voluntary.

The implemented SG cases were of varying difficulty and were capable of discriminating between varying surgical expertise. A total of 121 (92.4%) out of 131 participants selected the correct organ system at the initial assessment (Figure 2). When other optional specification levels were used, the result was predominantly correct (correct specification of complication mechanism: 77/88, 88%; Figure 3; correct specification of exact complication: 29/33, 88%; Figure 4).

All participating experts and board-certified surgeons correctly selected the suspected organ system after the initial patient presentation (Figure 2), demonstrating that case presentations provided sufficient information for surgical experts to conclude relevant details about the virtual case. In contrast, medical students and nonphysician caregivers selected incorrect organ systems more frequently. This demonstrates that varying surgical expertise was mirrored by the differences in correct case interpretation. The final diagnoses given at the end of the game also correlated with the participants’ level of training. In the final step of each case, physicians gave the correct diagnosis in all cases (Multimedia Appendices 7-12).

Based on the results of medical students and other staff, the cases varied in difficulty. The COVID-19 and wound infection (Multimedia Appendix 11) case was ultimately correctly interpreted by all participants, and most participants correctly diagnosed the virtual patients with stroke (16/19, 84%; Multimedia Appendix 8) and ileus (9/10, 90%; Multimedia Appendix 10). In contrast, sentinel hemorrhage (Multimedia Appendix 12), POPF (Multimedia Appendix 9), and 2 pancreatic surgery-specific complications were misspecified by 67% (12/18) and 43% (6/14) participants, respectively, with marked differences between physicians and other participant groups.

**Figure 5:**
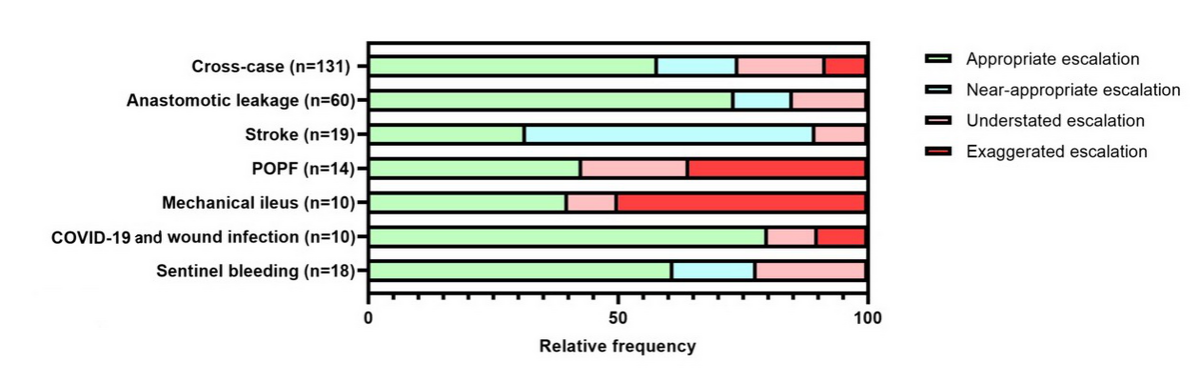
Case-specific evaluation of therapy escalation. In order to analyze the appropriateness of management, 1 therapeutic measure was defined as crucial for each serious gaming case based on valid guidelines. POPF: postoperative pancreatic fistula.

Figure 5 summarizes the case-specific therapy escalation. Overall, the assessment of therapy escalation led to particular difficulties in case 3 (POPF) and 4 (mechanical ileus). Case 5 (COVID-19 and wound infection), on the other hand, was correctly assessed by the great majority (17/19, 89%) of participants.

Multimedia Appendices 13-18 display the case-specific therapy escalation stratified by occupational and educational group.

### SG Is Accepted by Health Care Professionals

**Figure 6:**
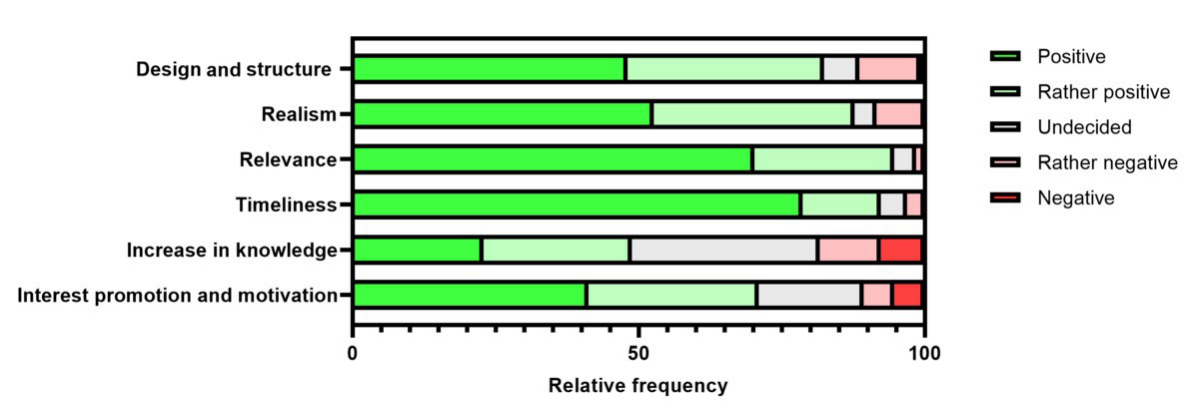
Results of the noncomparative validity and usefulness analysis. After completing a serious gaming case, participants were asked to rate it across several dimensions using a 5-point scale.

The goal of the VUA was to determine how instructive and user-friendly participants perceived the case-based SG application to be. Overall, design, structure, realism, relevance, and timeliness were predominantly rated positively (Figure 6). Interest promotion and motivation for autodidactic workup and estimated increase in knowledge were rated lower compared to the aforementioned dimensions. The detailed results of the VUA are summarized in Multimedia Appendix 19.

Most participants perceived case-based SG to be superior to conventional lecture-based learning and teaching formats (training courses, lectures, and seminars) in terms of increasing problem-solving skills (53/131, 41% perceived it superior; 49/131, 37% perceived it rather superior), self-reflection (50/131, 38% perceived it superior; 52/131, 40% perceived it rather superior), and usability and applicability (55/131, 42% perceived it superior; 49/131, 37% perceived it rather superior). Overall, 74 (57%) out of 131 participants perceived the SG application to be superior (26/131, 20% perceived it superior; 48/131, 37% perceived it rather superior) to these conventional teaching formats with regard to the gain of theoretical competencies, while 26 (20%) out of 131 participants did not see any difference. Overall, the case-based SG was rated “good” (“2”) or better in 78% (102/131) of cases (Multimedia Appendix 19).

## Discussion

### Principal Findings

This study presents a new didactic implementation of case-based learning (CBL) as a SG for medical education in the field of identification of surgical complications and validates this implementation in a representative cohort of participants with varying medical experience and expertise. Overall, the case-based SG format was well-accepted, and the majority of participants rated the platform as superior to traditional formats in terms of problem-solving skills, self-reflection, usability and applicability.

### Comparison to Previous Work

Postoperative complications are a part of the perioperative process that can only be prevented to a limited extent [9]. Timely recognition and straightforward treatment initiation are crucial to optimizing patient outcomes [2]. One limitation of conventional didactic methods lies in the poor transfer of theory into practice [30]. SG has the potential to markedly improve this transfer by teaching medical decision-making, training a systematic and complete approach, building up automatisms, and transferring these into a virtual action [31].

Complications after surgical interventions are common and closely linked to higher morbidity and mortality [2,3]. Despite knowledge about predisposing factors and early warning signs, clinical management guidelines generally leave room for individualized decisions that take clinical circumstances into consideration. In terms of such diagnostic and therapeutic decisions (ie, diagnostic computer tomography scan or application of antibiotics), a recent large-scale study [32] has compared a decision-making algorithm to standard care in the context of postoperative complications after pancreatic resection. Here, the algorithm-directed initiation of diagnostic and therapeutic measures resulted in a significantly reduced 90-day mortality rate, indicating that logic-triggered decision-making has the potential to improve clinical outcomes in postoperative care. The presented case-based SG platform could contribute to an integration of such knowledge and “quasi-algorithmic” decision-making into medical education, which could result in improved identification of early warning signs of serious complications after major abdominal surgery.

In aviation and military training, simulations (ie, flight simulators) have been an integral part of training curricula for over a century [33]. Adapted to the growing demand for digital and combined teaching concepts [34], the interest in SGs is currently growing in the field of medicine too [12]. Cases present a combination of theory and practice and can prepare users for clinically acute and challenging situations, with growing evidence suggesting the effectiveness of CBL [35,36]. Medical SGs address different specialties (surgery [37], internal medicine [38], etc) and target groups (patients [39], physicians [40], nurses [41], and students [18]) and vary regarding the type of application (web-based [42], virtual, or augmented reality [43]). To date, no comparable case-based SG for complication management in surgery is available. Existing works in the field of surgery mostly aim at training laparoscopy or other specific practical skills [37] and focus less on cases requiring complex multilevel management.

SG facilitates training under near-realistic conditions without the risk of harming patients [44]. Moreover, it has the potential to raise awareness about medical errors and, therefore, ultimately, reduce health care use and costs. Specific and rare medical scenarios are reproducible, which is challenging to teach in theoretical curricula. The strengths of SG lay in building up strategic and analytical skills and to train decision-making. As such, better attitudes toward learning and increased enthusiasm for learning were reported in individuals taught with SG in comparison to those taught with a written script [36,45].

The proposed platform does not impose any requirements for the end device, facilitating mobile, time- and place-independent, and inexpensive usage. In contrast, existing SG applications often rely on equipment that is prone to failure, expensive to develop and implement, and has limited scalability and availability [46]. Our overall results add to the growing body of evidence that CBL and SG have the potential to increase learning fun, which are important precursors for motivation and success [47,48]. Upscaling of the platform through transfer into other medical specialties or inclusion of other professional contexts of patient care, as well as an adaptation to rapidly updating medical evidence, are possible extensions of this work that are being investigated in follow-up trials.

In line with these findings, participants perceived the proposed SG application to be superior to traditional learning formats such as lectures with regard to gaining clinical and problem-solving skills and empowerment for self-reflection as well as higher joy in learning.

### Strengths and Limitations

The study results suggest that case-based digital SG could be a relevant addition to existing learning formats in the context of multimodal learning in medical education. The major strengths of the proposed SG application are its realism, topicality, and uncomplicated applicability and handling. The only dimensions of the VUA in which the SG platform was rated positive or superior in less than 50% of the cases were those of estimated increase in knowledge gain. These lower ratings in terms of subjectively perceived learning gains present a weakness of the SG format, which may be partially inherent to the methodology used. Rather than assessing concrete knowledge acquisition, we explored the subjective impact on learning. Future studies may gauge SGs’ impact on learning through diverse methods, such as multiarm interventions comparing SG with traditional approaches and long-term assessments including multiple-choice questions, clinical scenarios, simulator-based performance, practical exams, and Objective Structured Clinical Examinations. Still, learning is characterized by an interplay of different modalities that complement each other.

The limitations of this study are mostly related to the study design. First, the generalizability of the results is limited since participation was voluntary. As a consequence, the validation of the presented case-based SG platform primarily included interested individuals. Due to the voluntary participation, the subgroup sizes were variable and, in some cases, small, rendering subgroup analyses impossible. These sources of bias could be eliminated through other modes of inclusion, that is, cohort inclusion in the context of mandatory classes or a cross-sectional study with mandatory participation, improving the representativity and homogeneity of subgroups. Second, the representation of selectable diagnostic and therapeutic measures in the form of a finite list could have introduced bias, as participants could be guided in both a favorable (ie, toward correct measures) and an unfavorable direction (ie, toward wrong measures). In the future, enhancement could be achieved by substituting drop-down menus with free-text fields. These would facilitate more individual responses without the risk of influencing participants’ decision-making process. The results would therefore be more likely to mirror the unbiased thought process, but this process would make evaluation substantially more challenging. To provide quick and reliable feedback for free-text fields within a game, artificial intelligence–based language processing methods could be integrated to filter and categorize the free-text responses in terms of content [49,50], providing respective feedback. Additionally, a human expert review process could be connected with such automated feedback, which could be used in the case of difficulty classifying certain answers. Third, realistic conceptualization of cases was time- and labor-intensive and required an expert review process to guarantee case quality. Simplifying this procedure could be accomplished by implementing a standardized and user-friendly input interface for health care professionals, coupled with a structured and straightforward review process to guarantee medical accuracy and relevance. Third, our platform, similar to most other SGs in medical education, primarily analyzes knowledge gain, skill enhancement, attitudes, and satisfaction within a testing scope [12]. Future studies should venture an implementation attempt into real teaching with the aim to prove the effectiveness and clinical impact of the format and to map a learning curve after a longer period of participation.

Despite these limitations, this study introduces an innovative, scalable combination of case-based learning and SG with high educational potential, particularly in the context of clinical decision-making in the context of complication management in pancreatic and colorectal surgery.

### Future Directions

Until now, medical SGs, including our platform, primarily analyze knowledge gain, skill enhancement, attitudes, and satisfaction within a testing scope [12]. Future studies should venture an implementation attempt into real teaching with the aim to prove the effectiveness and clinical impact of the format and to map a learning curve after a longer period of participation. To boost knowledge gained through SG, various extensions, like stronger game characteristics, could potentially be integrated into these continuing studies, such as a coaching simulated senior physician to request advice from during the game.

### Conclusion

This study explores the use of SG in medical education, particularly for teaching surgical complication management. Using a desktop-based SG application, we assessed its effectiveness and acceptance among health care professionals. The findings reveal various strengths of the proposed case-based SG platform. The application presents real postoperative complications, allowing users to enhance their decision-making skills without risk. This approach enhances early complication identification, which could ultimately contribute to the sensitization of caregivers and better patient care and outcomes after abdominal surgery.

## Appendix

Multimedia Appendix 1 Measure categories and individual measures did not differ between cases and stages.

Multimedia Appendix 2 Initial serious gaming case presentations and ideal serious gaming case pathways with appropriate therapy escalation in bold face and near-appropriate therapy escalation in last row.

Multimedia Appendix 3 Anonymized participant data.

Multimedia Appendix 4 Average duration of serious gaming cases about age group.

Multimedia Appendix 5 Average duration of serious gaming cases about professional group or educational level.

Multimedia Appendix 6 Average duration of serious gaming cases about selected case.

Multimedia Appendix 7 Interprofessional evaluation of the final suspected diagnosis. Case: anastomotic leakage.

Multimedia Appendix 8 Interprofessional evaluation of the final suspected diagnosis. Case: stroke.

Multimedia Appendix 9 Interprofessional evaluation of the final suspected diagnosis. Case: postoperative pancreatic fistula (POPF).

Multimedia Appendix 10 Interprofessional evaluation of the final suspected diagnosis. Case: mechanical ileus.

Multimedia Appendix 11 Interprofessional evaluation of the final suspected diagnosis. Case: COVID-19 or wound infection.

Multimedia Appendix 12 Interprofessional evaluation of the final suspected diagnosis. Case: sentinel bleeding.

Multimedia Appendix 13 Evaluation of correct therapy escalation (in brackets) by occupational group. Case: anastomotic leakage (laparotomy).

Multimedia Appendix 14 Evaluation of correct therapy escalation (in brackets) by occupational group. Case: stroke (lysis therapy [thrombolytics, eg, alteplase]).

Multimedia Appendix 15 Evaluation of correct therapy escalation (in brackets) by occupational group. Case: POPF (Computed tomography + intervention: drainage system). POPF: postoperative pancreatic fistula.

Multimedia Appendix 16 Evaluation of correct therapy escalation (in brackets) by occupational group. Case: mechanical ileus (stoma extension on fascia level).

Multimedia Appendix 17 Evaluation of correct therapy escalation (in brackets) by occupational group. Case: COVID-19 or wound infection (medical history and clinical examination).

Multimedia Appendix 18 Evaluation of correct therapy escalation (in brackets) by occupational group. Case: sentinel bleeding (laparotomy).

Multimedia Appendix 19 Complete validity and usefulness analysis.

## Abbreviations

CBL: case-based learning
POPF: postoperative pancreatic fistula
REDCap: Research Electronic Data Capture
SG: serious gaming (or serious game)
VUA: validity and usefulness analysis

## Data Availability

The case descriptions and ideal clinical pathways are available in Multimedia Appendix 2. Participant data gathered in the context of this work are available in Multimedia Appendix 3.
