# Supplementary material for: Case-Based Serious Gaming for Complication Management in Colorectal and Pancreatic Surgery: Prospective Observational Study
Source: JMIR Serious Games. 2023 Nov 9;11:e44708. doi: 10.2196/44708 (PMC10667978; doi:10.2196/44708)
Supplement: Multimedia Appendix 1 [file games_v11i1e44708_app1.docx]

Multimedia Appendix 1: Measure categories and individual measures did not differ between cases and stages.

| **Measure categories** | **Individual measures** |
| --- | --- |
|  |  |
| Clinical examination / history | - Clinical examination / history (range: complete clinical examination with digital rectal examination at discretion) |
| Scores | - Wells-Score - National Institutes of Health Stroke Scale - Glasgow coma score - Mini-Mental-Status |
| Functional / metabolic tests | - (Ergo)spirometry - Lung function examination - Electrocardiogram - Electroencephalography - Positron emission tomography |
| Monitoring vital signs / blood | - Monitoring of vital parameters (blood pressure, pulse, oxygen saturation) - Transfusion of blood or blood components |
| Wounds / stoma / foreign material | - Stoma care and stoma protocol - Surgical wound care - Wound checks - Drainage removal - Removal of the indwelling catheter - Removal of the vascular access - Drainage control: appearance, flow rate and enzyme activity |
| Medical consults / transfer | - Intensive care (incl. monitoring, drug and interventional therapy depending on the findings) - Pulmonology / COVID-19 ward - Cardiology (e.g. echocardiography, co-assessment of pathological electrocardiogram findings) - Neurology / Stroke Unit - Neurosurgery - Interventional (neuro-)radiology - Dermatology - Gynecology - Urology - Clinical infectiology - Isolation in infectious diseases |
| Laboratory / Pathology / Microbiology | - Venous blood test - Blood gas analysis - Venous blood analysis + Blood gas analysis - Microbiology: blood culture aerobic + anaerobic - Urine stick - Laboratory / microbiology: urine examination (microscopy, urine sediment, urine culture) - Microbiology: nasopharyngeal smear - Microbiology: wound swab - Laboratory: examination of drainage fluid |
| Sonography and sonography-assisted interventions | - Sonography of the thorax (scope: pleura, pleural effusion?, pneumothorax?) - Sonography of the abdomen (scope: organ assessment, abdominal vessels) - Extended Focussed Assessment with Sonography for Trauma - Sonographically assisted puncture: pleural effusion + examination - Sonographically assisted puncture: pericardial effusion + examination - Sonographically assisted puncture: ascites + examination - Sonographical follow-up |
| X-ray | - X-ray thorax - X-ray abdomen - X-ray skull - Esophageal passage |
| CT and CT-guided interventions | - CT thorax / abdomen / pelvis native - CT thorax / abdomen / pelvis with i.v. contrast - CT thorax / abdomen / pelvis with i.v. and oral / rectal contrast - CT skull native - CT skull + supraaortic CT angiography - CT angio: pelvic-leg arteriography - CT + intervention: drainage - CT + intervention: coiling / embolization of bleeding source - CT follow-up |
| MRI / MRI-assisted interventions | - MRI thorax / abdomen / pelvis native - MRI thorax / abdomen / pelvis with i.v. contrast - MRI thorax / abdomen / pelvis with i.v. and oral / rectal contrast - MRI skull - MRI skull + supraaortic angiography - MRI Angio: pelvic-leg arteriography - MRI + intervention: drainage system - MRI + intervention: coiling / embolization of bleeding source - MRI + intervention: pleural effusion puncture + examination - MRI + intervention: pericardial effusion puncture + examination - MRI + intervention: ascites puncture + examination |
| Discontinue medication | - Anticoagulation - Antibiotics - Antihypertensives - Discontinuation of all medications |
| Set medications | - Non-opioid analgesics / non-steroidal anti-inflammatory drugs (per os) - Non-opioid analgesics / non-steroidal anti-inflammatory drugs (i.v.) - Opioid analgesics (per os) - Opioid analgesics (i.v.) - Antihypertensives - Volume therapy / electrolyte balancing - Antibiotics without previous antibiogram - Antibiotics with previous antibiogram - Blood thinners - Lysis therapy (thrombolytics, e.g. alteplase) - after prior critical evaluation - Prokinetics - Antiemetics - Diuretics - Insulin - Glucose - Somatostatin analogues (octreotide) |
| Nutrition / feeding tube | - Parenteral nutrition - Insertion of a gastric tube - Rectal enema - Stoma enema - Gentle nutrition |
| Endoscopy / invasive examinations (+intervention) | - Cardiac catheterization, with intervention if necessary - Esophagogastroduodenoscopy, with intervention if necessary - Colonoscopy, with intervention if necessary - Rectoscopy, with intervention if necessary - Esophagogastroduodenoscopy + colonoscopy, with intervention if necessary - ERCP, with intervention if necessary - MRCP, with intervention if necessary - PTC / PTCD - Skull: Mechanical recanalization after initial lysis therapy |
| Re-surgery / laparoscopy / laparotomy | - Explorative laparotomy with procedure according to findings - Explorative laparoscopy with procedure according to findings - Superficial skin incision / puncture: abscess / hematoma evacuation - Stoma extension to fascia level |
