# Supplementary material for: Case-Based Serious Gaming for Complication Management in Colorectal and Pancreatic Surgery: Prospective Observational Study
Source: JMIR Serious Games. 2023 Nov 9;11:e44708. doi: 10.2196/44708 (PMC10667978; doi:10.2196/44708)
Supplement: Multimedia Appendix 2 [file games_v11i1e44708_app2.docx]

Multimedia Appendix 2: Initial serious gaming case presentations (A) and ideal serious gaming case pathways with appropriate therapy escalation in **bold face** and near-appropriate therapy escalation in last row (B). Abbreviations: COVID-19: Coronavirus disease. POPF: Postoperative pancreatic fistula.

|  | **Anastomotic leakage** | **Stroke** | **POPF** | **Ileus** | **COVID-19 / wound infection** | **Sentinel bleeding** |
| --- | --- | --- | --- | --- | --- | --- |
|  |  |  |  |  |  |  |
| **Data (gender, age, postoperative day (POD))** | M / 62 years / POD: 4 / Surgery: stoma reversal. | M / 83 years / POD: 4 / Surgery: Right hemicolectomy. | F / 65 years / POD: 4 / Surgery: Distal pancreatectomy. | M / 87 years / POD: 2 / Surgery: left hemicolectomy with protective ileostomy. | F / 88 years / POD: 14 / Surgery: total rectal extirpation. | F / 71 years / POD: 7 / Surgery: Whipple. |
| **Current symptoms** | Stool is noticed in the drainage. Abdomen: bowel sounds in all four quadrants, guarding in left upper/lower abdomen, skin is unremarkable. No heart murmurs. Regular breath sounds. | Patient is no longer speaking and is dazed. Nurse has checked and secured vital signs, i.v. access is in place. It is unclear what mental status the patient came in. | Elevated temperature for the first time (39.4°C). Patient also presents in a worsened general condition, shows guarding in the upper abdomen, and states abdominal pain radiating to the back. Blood pressure: 105/70, pulse slightly elevated. The surgical scar looks unremarkable at first glance. Renal positional palpitation appears to be present. The patient no longer has an indwelling catheter. | Patient presents today with abdominal pain and nausea. An image of the abdomen is sent to you: abdomen is distended, stoma bag is empty. The patient has already started to build up his diet. The surgical scar looks good, drainage fluid is clear. | Family physician re-admits patient with rising CRP. Wound looks infected. Other symptoms include fever, headache, and circulatory problems. A COVID-19 rapid test was done in the emergency room and negative. PCR result is pending. No heart murmurs. Basal attenuated breath sounds. | Drainage was already tinged with blood over the weekend. Today (monday) the drainage fluid appears bloody again. Deteriorated general condition, circulatory dysfunction. The surgical scar looks good, the patient expresses diffuse abdominal pain. |
|  |  |  |  |  |  |  |
| **Acute management** | CT thorax / abdomen / pelvis with i.v. and oral / rectal contrast | CT skull + supraaortic CT angiography | CT thorax / abdomen / pelvis with i.v. contrast | X-ray / CT abdomen | Surgical wound care | CT thorax / abdomen / pelvis with i.v. contrast |
|  | Venous blood test | Option 1: Medical consult: Neurology | Venous blood test | Insertion of a gastric tube | Venous blood test | Transfusion of blood or blood components |
|  | Monitoring of vital parameters | Option 2: Combination: Clinical examination / history + NIHSS | Non-opioid analgesics / non-steroidal anti-inflammatory drugs (i.v.) | Clinical examination + history | **Clinical examination + history** | Discontinuation of anticoagulation |
| **Definitive management** | Explorative laparoscopy with procedure according to findings | Transfer neurology / stroke unit | **CT + intervention: drainage system** | **OP: stoma extension on fascia level** | X-ray thorax | CT + intervention: coiling / embolization of source of bleeding |
|  | Antibiosis (without antibiogram) | Medical consult: interventional (neuro-)radiology | Parenteral nutrition | Venous blood test | Transfer to pulmonology / COVID-19 ward | Transfusion of blood or blood components |
|  |  | **Lysis therapy (thrombolytics, e.g., alteplase)** | Antibiotics with previous antibiogram |  | Antibiotics with previous antibiogram |  |
| **Longterm management** | **Exploratory laparotomy with procedure according to findings** | Transfer to Neurology / Stroke Unit | Sonographical follow-up | Stoma care + stoma protocol | Medical consult: Clinical infectiology | **Exploratory laparotomy with procedure according to findings** |
|  | Transfer to intensive care unit | Medical consult: Cardiology | Drainage control: flow rate, enzyme activity |  | Wound controls | Transfer to intensive care unit |
|  |  | Monitoring of vital parameters | Removal of drainage |  |  |  |
| **Near-appropriate therapy escalation** | Exploratory laparoscopy with procedure according to findings | Medical consult: Neurology |  |  |  | Exploratory laparoscopy with procedure according to findings |
|  | Background for the introduction of a further level in the case of anastomotic leakage and sentinel hemorrhage: Laparoscopy allows intraoperative therapy escalation to laparotomy, thus can be considered as near appropriate escalation.  Background for the introduction of a further level in the case of stroke: The neurological consult resulted in lysis therapy. Due to the lack of autonomy of the procedure, the selection of this measure is only rated as "near-appropriate". | | | | | |
