# Supplementary material for: Case-Based Serious Gaming for Complication Management in Colorectal and Pancreatic Surgery: Prospective Observational Study
Source: JMIR Serious Games. 2023 Nov 9;11:e44708. doi: 10.2196/44708 (PMC10667978; doi:10.2196/44708)
Supplement: Multimedia Appendix 4 [file games_v11i1e44708_app4.docx]

Multimedia Appendix 4: Average duration of serious gaming cases about age group.

| Age [years] | n | Average duration [hh:mm:ss] | *P*-value |
| --- | --- | --- | --- |
|  |  |  |  |
| <20 | 7 | 00:13:53 | *P*=.46 |
| 21-25 | 45 | 00:08:32 |  |
| 26-30 | 36 | 00:09:29 |  |
| 31-35 | 12 | 00:09:59 |  |
| 36-40 | 7 | 00:11:43 |  |
| 41-45 | 9 | 00:05:50 |  |
| 46-50 | 3 | 00:03:17 |  |
| 51-55 | 7 | 00:10:29 |  |
| 56-60 | 4 | 00:09:39 |  |
| 61-65 | 1 | 00:14:04 |  |
| Total | 131 | 00:11:46 |  |
