# Supplementary material for: Case-Based Serious Gaming for Complication Management in Colorectal and Pancreatic Surgery: Prospective Observational Study
Source: JMIR Serious Games. 2023 Nov 9;11:e44708. doi: 10.2196/44708 (PMC10667978; doi:10.2196/44708)
Supplement: Multimedia Appendix 5 [file games_v11i1e44708_app5.docx]

Multimedia Appendix 5: Average duration of serious gaming cases about professional group / educational level.

| Professional group / Education level | n | Average duration [hh:mm:ss] | *P*-value |
| --- | --- | --- | --- |
|  |  |  |  |
| Experts (chief and senior physicians) | 18 | 00:09:50 | *P*=.33 |
| Board-certified surgeons | 5 | 00:11:37 |  |
| Surgical residents | 14 | 00:11:06 |  |
| Medical students | 74 |  |  |
| Years 1-5 | 30 | 00:11:26 |  |
| Final year (year 6) | 44 | 00:12:20 |  |
| Nursing staff | 12 | 00:12:20 |  |
| Other clinical staff with patient contact | 8 | 00:15:01 |  |
