# Supplementary material for: Case-Based Serious Gaming for Complication Management in Colorectal and Pancreatic Surgery: Prospective Observational Study
Source: JMIR Serious Games. 2023 Nov 9;11:e44708. doi: 10.2196/44708 (PMC10667978; doi:10.2196/44708)
Supplement: Multimedia Appendix 6 [file games_v11i1e44708_app6.docx]

Multimedia Appendix 6: Average duration of serious gaming cases about selected case. Abbreviations: POPF: Postoperative pancreatic fistula, COVID-19: Coronavirus disease.

| Completed serious gaming cases | n | Average duration [hh:mm:ss] | *P*-value |
| --- | --- | --- | --- |
|  |  |  |  |
| Anastomotic leakage | 60 | 00:12:41 | *P*=.68 |
| Stroke | 19 | 00:10:28 |  |
| POPF | 14 | 00:10:01 |  |
| Mechanical ileus | 10 | 00:10:53 |  |
| COVID-19 / wound infection | 10 | 00:14:10 |  |
| Sentinel bleeding | 18 | 00:12:24 |  |
