# Supplementary figures and images for: Case-Based Serious Gaming for Complication Management in Colorectal and Pancreatic Surgery: Prospective Observational Study
Source: JMIR Serious Games. 2023 Nov 9;11:e44708. doi: 10.2196/44708 (PMC10667978; doi:10.2196/44708)

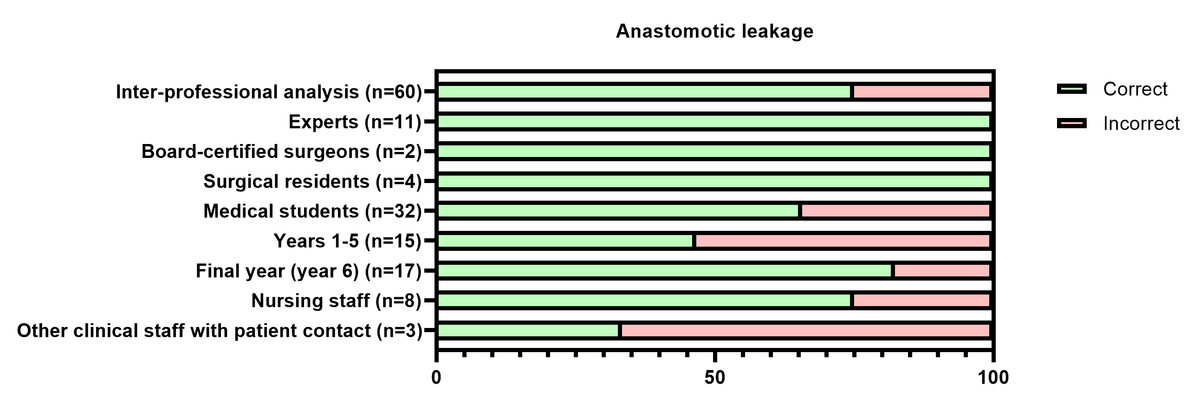

Supplement: Multimedia Appendix 7 [file games_v11i1e44708_app7.png]

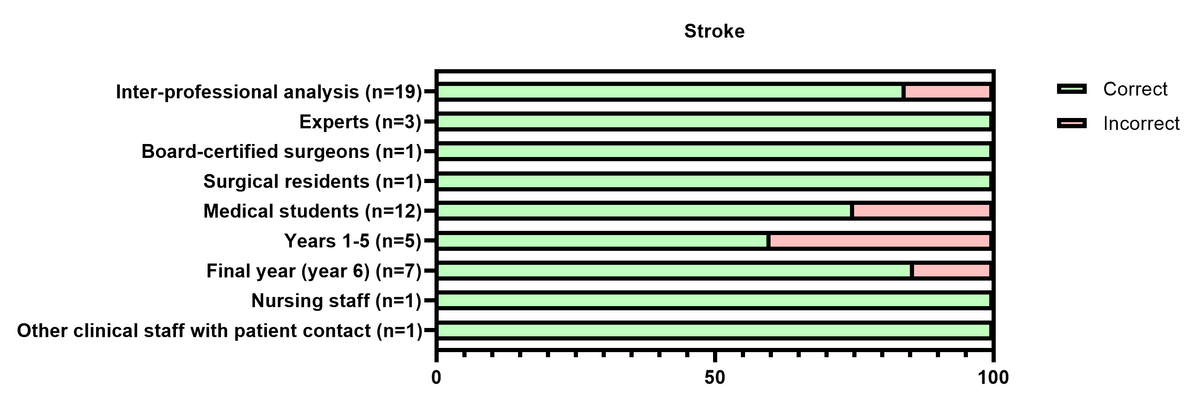

Supplement: Multimedia Appendix 8 [file games_v11i1e44708_app8.png]

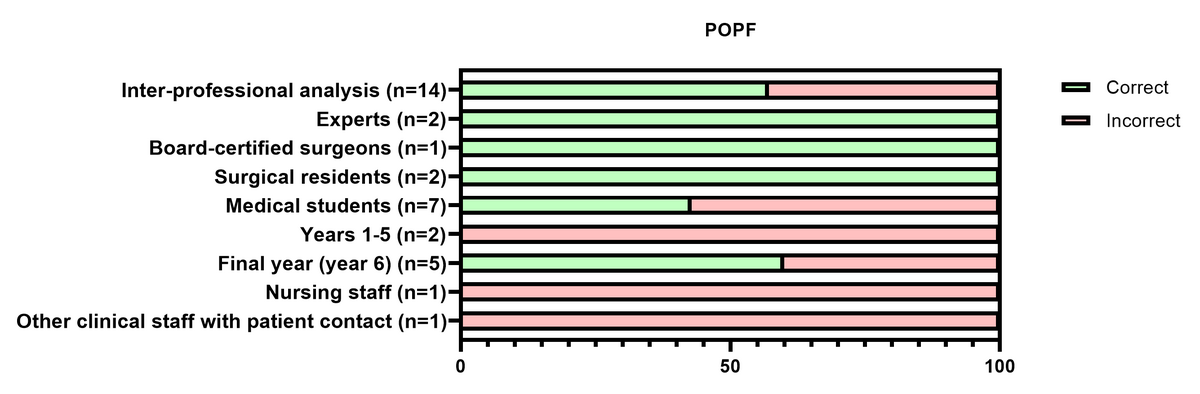

Supplement: Multimedia Appendix 9 [file games_v11i1e44708_app9.png]

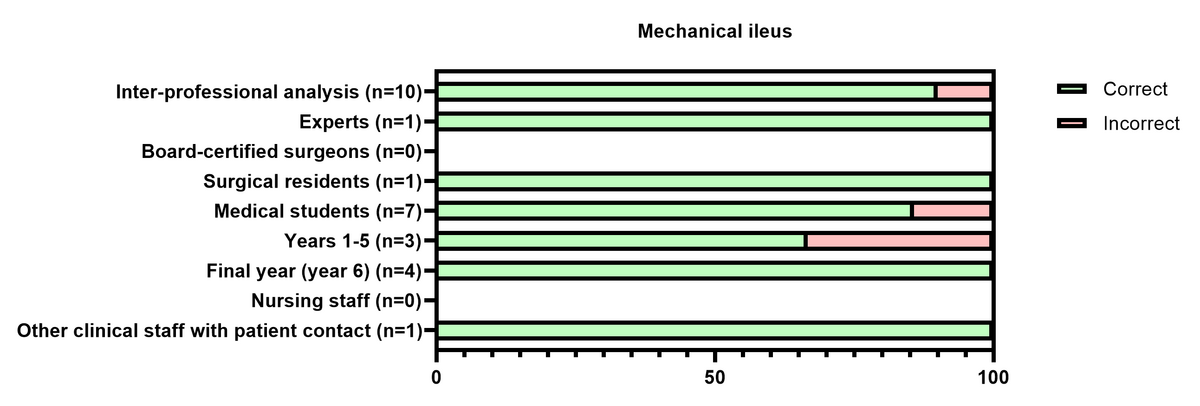

Supplement: Multimedia Appendix 10 [file games_v11i1e44708_app10.png]

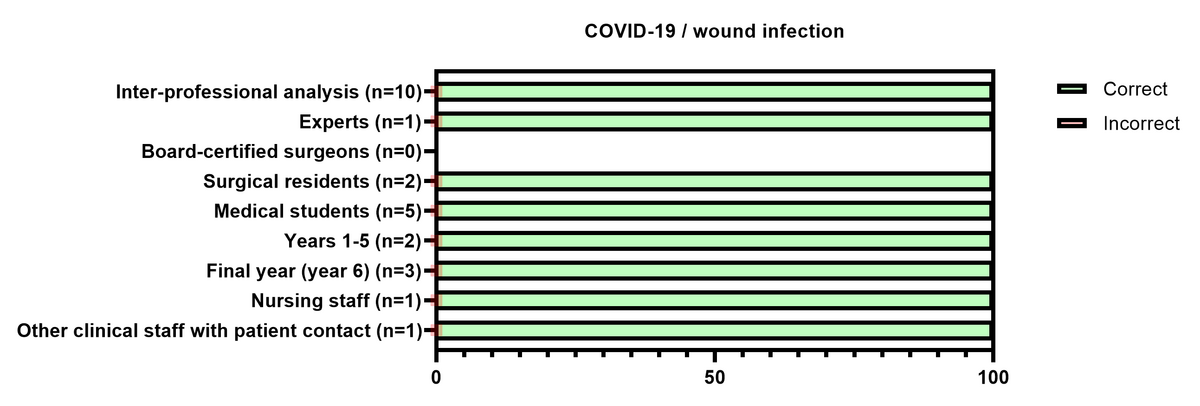

Supplement: Multimedia Appendix 11 [file games_v11i1e44708_app11.png]

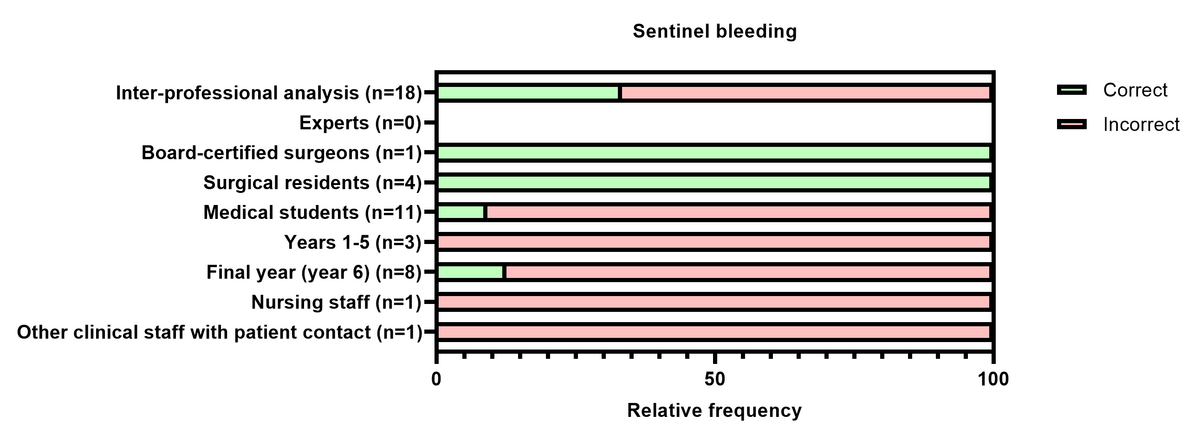

Supplement: Multimedia Appendix 12 [file games_v11i1e44708_app12.png]

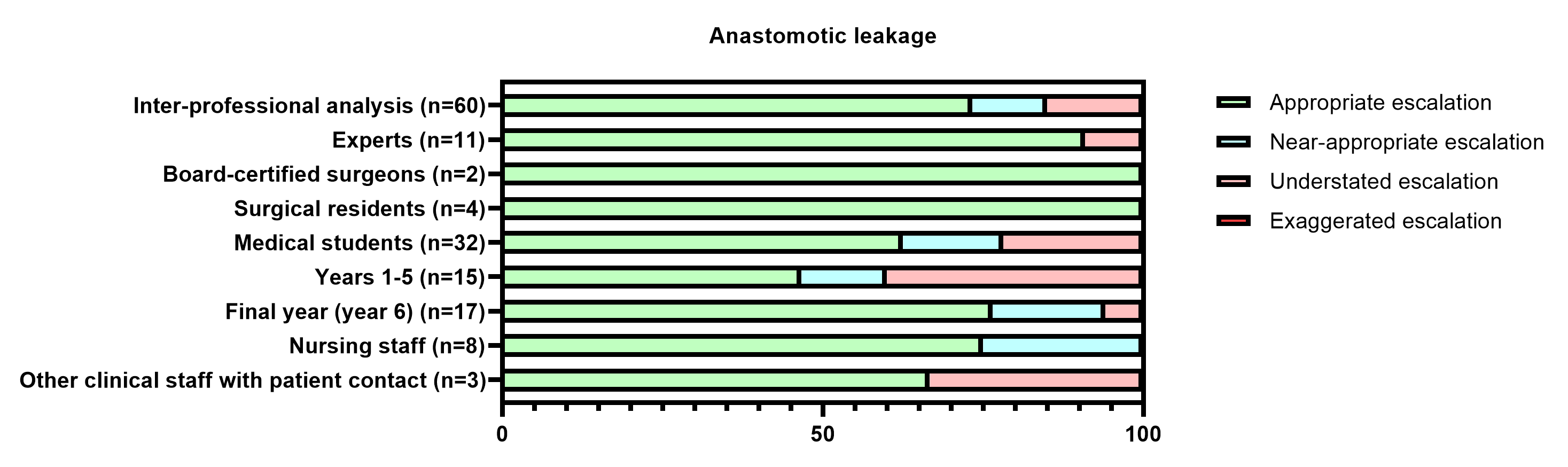

Supplement: Multimedia Appendix 13 [file games_v11i1e44708_app13.png]

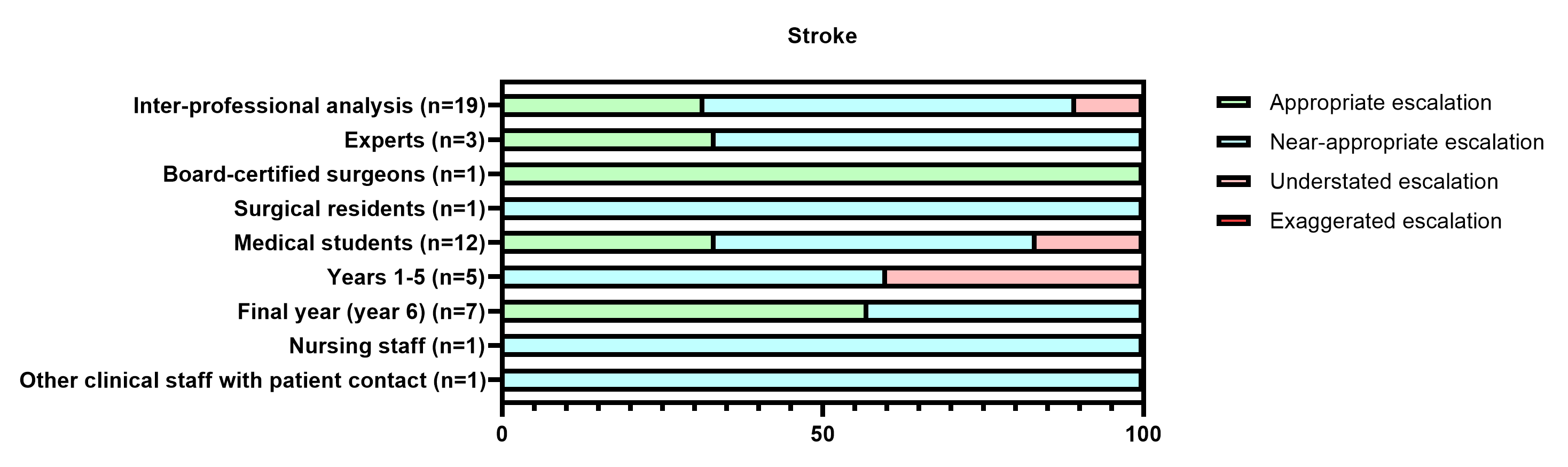

Supplement: Multimedia Appendix 14 [file games_v11i1e44708_app14.png]

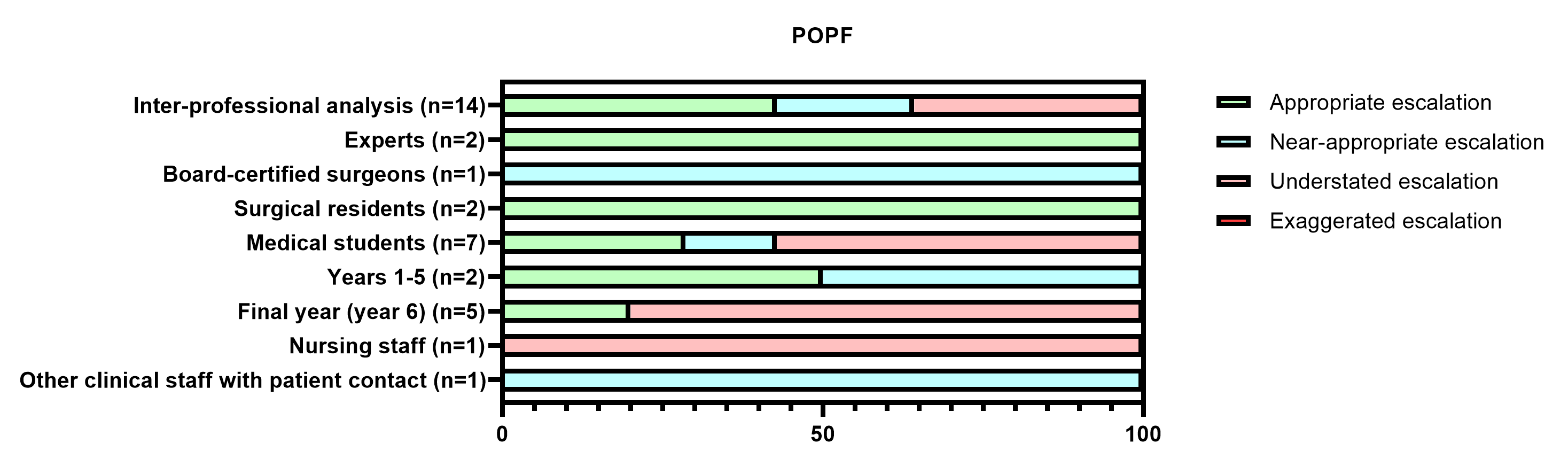

Supplement: Multimedia Appendix 15 [file games_v11i1e44708_app15.png]

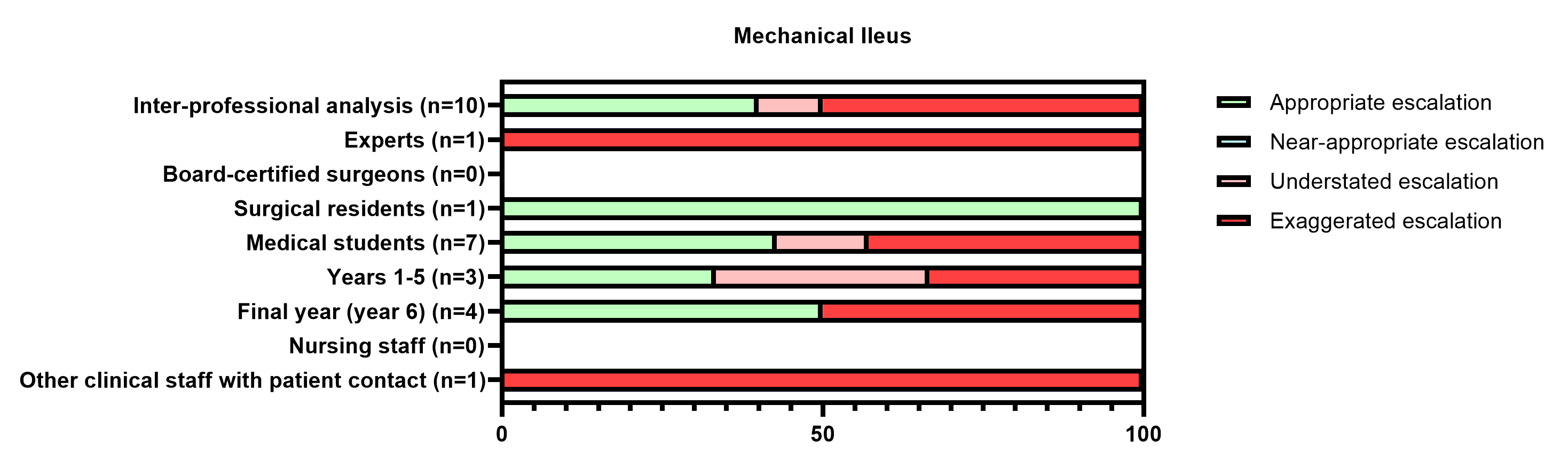

Supplement: Multimedia Appendix 16 [file games_v11i1e44708_app16.png]

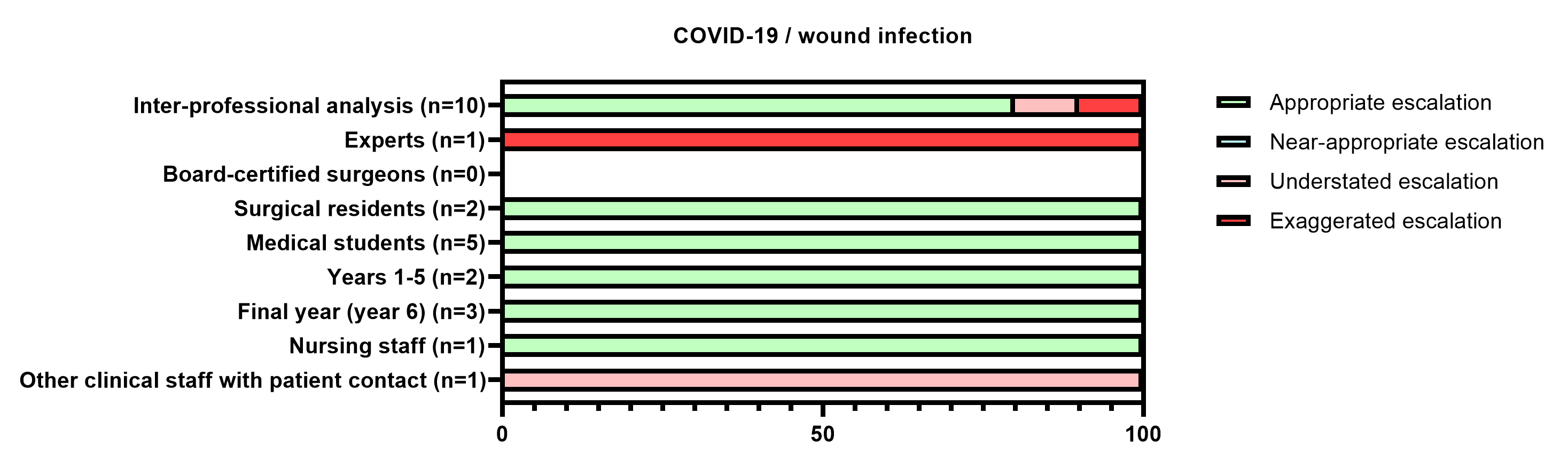

Supplement: Multimedia Appendix 17 [file games_v11i1e44708_app17.png]

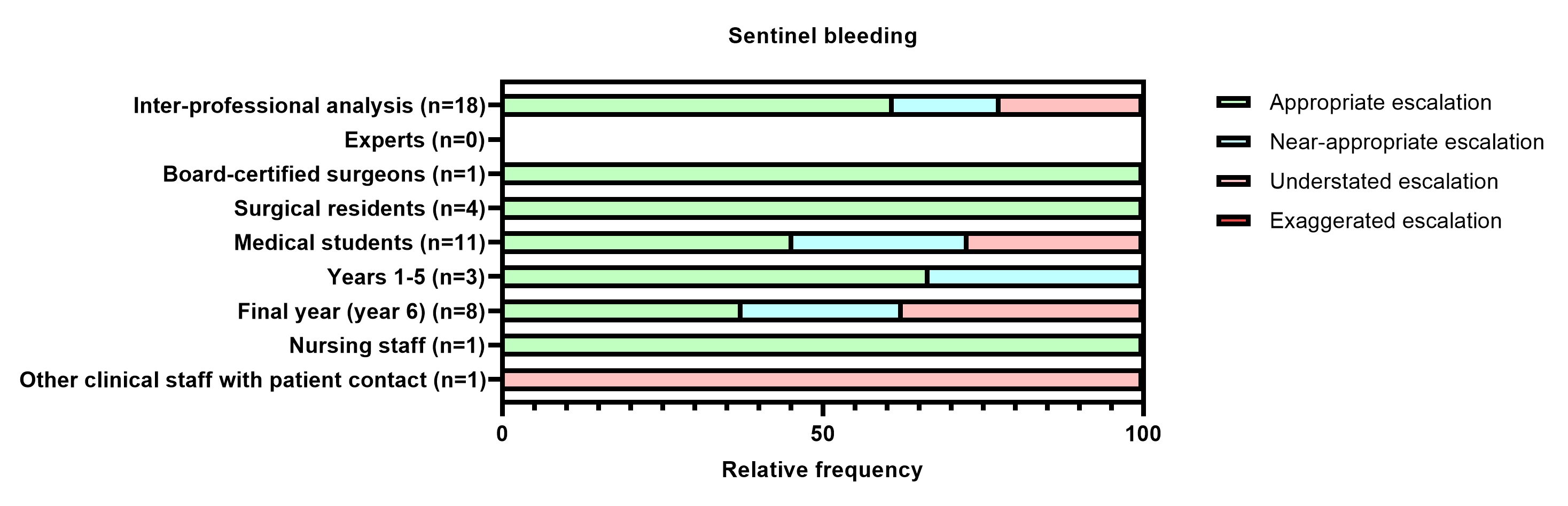

Supplement: Multimedia Appendix 18 [file games_v11i1e44708_app18.png]
