# Supplementary material for: Case-Based Serious Gaming for Complication Management in Colorectal and Pancreatic Surgery: Prospective Observational Study
Source: JMIR Serious Games. 2023 Nov 9;11:e44708. doi: 10.2196/44708 (PMC10667978; doi:10.2196/44708)
Supplement: Multimedia Appendix 19 [file games_v11i1e44708_app19.docx]

Multimedia Appendix 19: Complete Validity and Usefulness Analysis. Abbreviations: SG: Serious gaming / serious game.

| **Part one: Non-comparative analysis (1=true/high to 5=does not apply/low)** | | | | | |
| --- | --- | --- | --- | --- | --- |
|  | **1** | **2** | **3** | **4** | **5** |
|  |  |  |  |  |  |
| “The design and structure of the SG application was intuitive and clear, allowing for easy editing.” | 48% | 34% | 6% | 11% | 1% |
| “The case was presented appropriately in scope and form (text and image) so that it reflected reality within the bounds of possibility.” | 53% | 35% | 4% | 8% | 0% |
| “I perceive the relevance of the postoperative patient case to be...” | 70% | 24% | 4% | 2% | 0% |
| “The content of the SG was up to date.” | 79% | 14% | 4% | 3% | 0% |
| “I estimate my personal knowledge gain to be...” | 23% | 26% | 33% | 11% | 7% |
| “The SG application furthered my interest in the topic and motivated me to follow up.” | 41% | 30% | 18% | 5% | 5% |
|  |  | | | | |
| **Part two: Comparative analysis (1=SG is superior to 5=SG is inferior, 3=no difference)** | | | | | |
| "Gain in theoretical competencies after using the learning format." | 20% | 37% | 20% | 20% | 3% |
| "Gain in clinical skills after using the learning format." | 24% | 47% | 17% | 11% | 1% |
| "Learning with the learning format brings me joy and motivates me." | 55% | 27% | 8% | 10% | 0% |
| "Through the learning format, I can increase my problem-solving skills - that is: identifying a problem and approaching it in a structured way." | 41% | 37% | 9% | 12% | 1% |
| "The learning format enables me to self-reflect - in other words: to be able to assess my own performance." | 38% | 40% | 8% | 12% | 2% |
| "The learning format can reflect the current state of the art, providing optimal continuing education." | 27% | 32% | 26% | 15% | 0% |
| "The learning format is easy to use for me and impresses with its flexible applicability. I can well imagine using it regularly in my everyday life." | 42% | 38% | 9% | 9% | 2% |
|  |  | | | | |
| **Part three: Grading and knowledge gain (1=SG is superior to 5=SG is inferior, 3=no difference)** | | | | | |
| With what school grade would you rate the SG application (grade 1-6)? | 32% | 46% | 12% | 8% | 2% |
| Increase in knowledge compared to known theoretical formats | 15% | 37% | 23% | 23% | 2% |
| Increase in knowledge compared to known practical formats | 15% | 18% | 23% | 33% | 11% |
